# Comparative Effectiveness of Single vs Repeated Rapid SARS-CoV-2 Antigen Testing Among Asymptomatic Individuals in a Workplace Setting

**DOI:** 10.1001/jamanetworkopen.2022.3073

**Published:** 2022-03-18

**Authors:** Bradley A. Connor, Marina Rogova, Jefferson Garcia, Darshan Patel, Mara Couto-Rodriguez, Dorottya Nagy-Szakal, Michael Rendel

**Affiliations:** 1 Weill Cornell Medicine, New York, New York; 2 The New York Center for Travel and Tropical Medicine, New York; 3 The Reed Group, New York, New York; 4 Biotia, Inc, New York, New York; 5 Department Cell Biology, College of Medicine, SUNY Downstate Health Sciences University, New York, New York; 6 Mount Sinai School of Medicine, New York, New York; 7 Goldman Sachs Group Inc, New York, New York

## Abstract

This comparative effectiveness research study assesses the accuracy of single vs repeated antigen testing for diagnosis of COVID-19 among asymptomatic individuals in a workplace setting.

## Introduction

The gold standard for diagnostic testing for SARS-CoV-2 is real-time reverse transcriptase–polymerase chain reaction (RT-qPCR).^1^ Because of lag times in obtaining results and the expense of this diagnostic modality, rapid antigen testing has been frequently used for screening of asymptomatic populations instead.^2^ Several SARS-CoV-2 rapid antigen tests are available in the US with Emergency Use Authorization by the US Food and Drug Administration based on data from symptomatic patients.^3^ Although rapid antigen tests have been criticized for poor sensitivity and specificity when screening asymptomatic patients, a knowledge gap still remains regarding the utility of these tests for screening.^4^ Given the large cohort of individuals being screened by an international service company based in New York City, we sought to analyze the comparative effectiveness and estimated accuracy of an employee screening program using single vs repeated antigen tests compared with RT-qPCR among asymptomatic individuals.

## Methods

This retrospective comparative effectiveness research study followed the International Society for Pharmacoeconomics and Outcomes Research (ISPOR) reporting guideline. Antigen testing of midturbinate nasal swab specimens was performed by trained personnel using Sofia2 SARS Antigen Fluorescent Immunoassay (Quidel Corporation) (positive percent agreement [PPA]: 96.7%; negative percent agreement [NPA]: 100%), LumiraDX (Abbott) (PPA: 97.6%; NPA: 96.6%), and BinaxNow (Abbott) (PPA: 84.6%; NPA: 98.5%) rapid antigen tests from November 27, 2020, to October 21, 2021. Testing cadence was based on the participant’s work schedule, work location, and ability for testing. Individuals with any of the cardinal symptoms of COVID-19 were excluded from screening. Those with a positive antigen test result were offered a second antigen test of a nasal swab specimen within an hour of the first test result. Nasal swab specimens were also sent to a Clinical Laboratory Improvement Amendments–certified laboratory for confirmatory RT-qPCR testing using the Cepheid GeneXpert RT-qPCR assay. Estimated accuracy was calculated as the percentage of second antigen tests with a positive result for which the RT-qPCR test result was also positive. The protocol was classified as public health surveillance and not human participant research by a human participants advisor at the US Centers for Disease Control and Prevention National Center for Emerging and Zoonotic Infectious Diseases and was therefore exempt from institutional review board approval, with a waiver of informed consent.

## Results

A total of 179 127 participants underwent testing and were included in the analysis. The median age was 36 years (range, 18-65 years); 58% were male, 36% were female, and gender was unknown for 6%.

A total of 179 127 rapid SARS-CoV-2 antigen tests were performed, with a 0.35% positivity rate (623 positive antigen test results) between November 2020 and October 2021 (Table). Of 623 total positive test results, 238 (38%) were confirmed to be true positive and 385 (62%) false positive by RT-qPCR. Of the 623 tests with positive results, 569 (91%) were followed by a second rapid antigen test. Of 224 sets of tests with concordant results (2 separate but consecutive antigen tests with positive results), RT-qPCR results were positive for 207 (92%). When the result of the first antigen test was positive and the result of the second antigen test was negative (n = 345), RT-qPCR results were negative for 328 (95%). The overall estimated accuracy of a second antigen test was 94%. The estimated accuracy by month, monthly trends, and comparison with New York City community rates are shown in the Figure.

**Table.** Overall Daily SARS-CoV-2 Test Results From an Employee Screening Program From November 27, 2020, to October 21, 2021

Date	Total tests^a^	Tests, No. (%)^b^			
		Antigen test result		Antigen test result vs RT-qPCR test result	
		Negative	Positive	True positive	False positive
2020					
November	429	424 (98.83)	5 (1.17)	2 (0.47)	3 (0.70)
December^c^	5454	5415 (99.28)	39 (0.72)	15 (0.28)	24 (0.44)
2021					
January	14 168	14 112 (99.60)	56 (0.40)	21 (0.15)	35 (0.25)
February	21 508	21 412 (99.55)	96 (0.45)	26 (0.12)	70 (0.33)
March	31 756	31 614 (99.55)	142 (0.45)	51 (0.16)	91 (0.29)
April	30 642	30 553 (99.71)	89 (0.29)	35 (0.11)	54 (0.18)
May	25 134	25 059 (99.70)	75 (0.30)	7 (0.03)	68 (0.27)
June	8361	8320 (99.51)	41 (0.49)	3 (0.04)	38 (0.45)
July	5595	5583 (99.79)	12 (0.21)	12 (0.21)	0
August	8148	8120 (99.66)	28 (0.34)	28 (0.34)	0
September	26 991	26 951 (99.85)	40 (0.15)	38 (0.14)	2 (0.01)
October	941	941 (100)	0	0	0
Total	179 127	178 504 (99.65)	623 (0.35)	238 (0.13)	385 (0.21)

Abbreviation: RT-qPCR, real-time reverse transcriptase–polymerase chain reaction.

^a^ Missing, pending, and invalid test results were excluded from the study.

^b^ The percentage was calculated with the total number of tests performed during the month as the denominator.

^c^ Results of 46 tests performed between December 24 and 31, 2020, were excluded owing to tests having dual strips.

**Figure.**
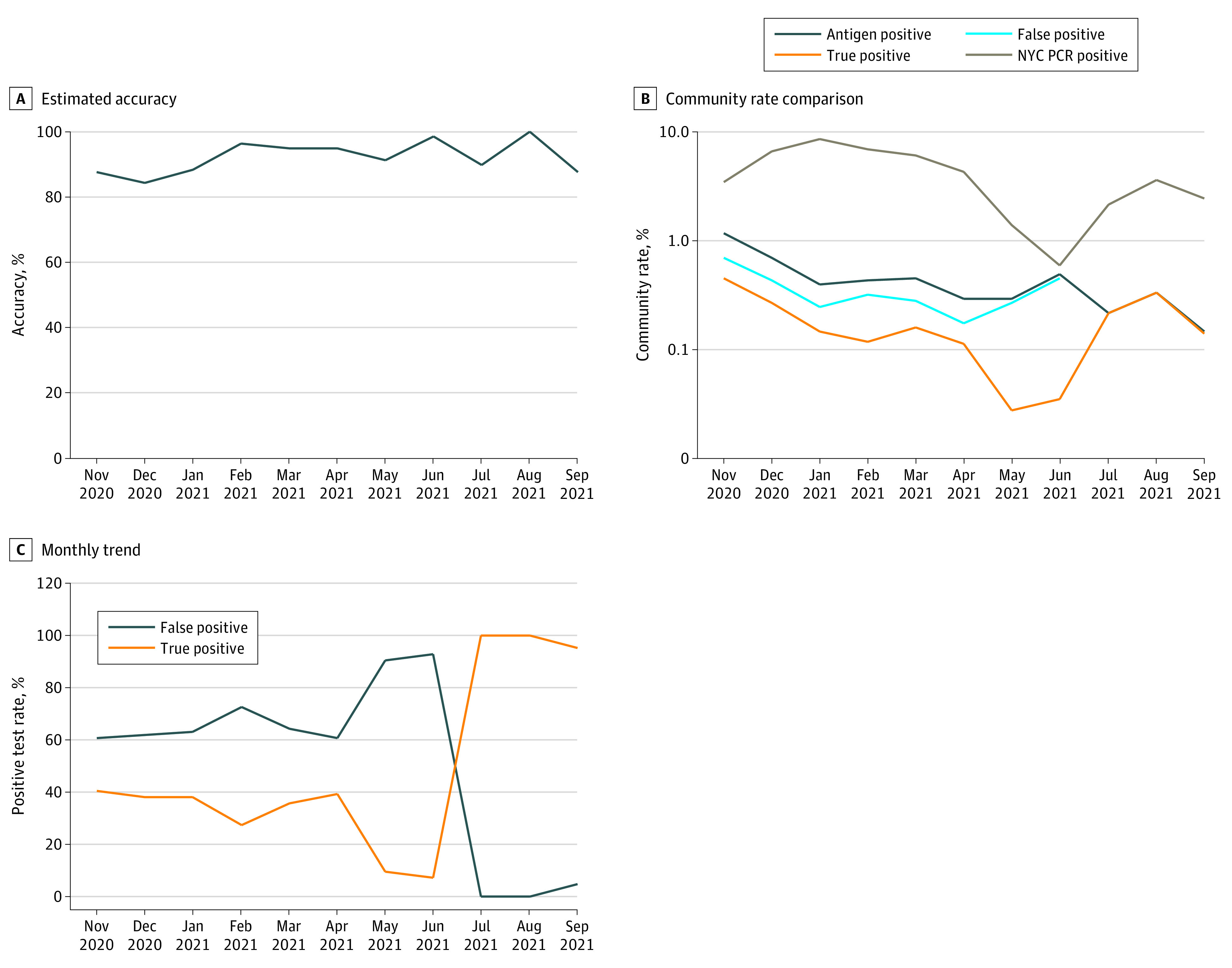
Estimated Accuracy of SARS-CoV-2 Antigen Tests by Month and Comparison of Test Positivity Rates and Monthly Trends in Rates With New York City (NYC) Community Infection Rates A, Estimated accuracy was calculated as the percentage of second antigen tests with a positive result for which the real-time reverse transcriptase–polymerase chain reaction (RT-qPCR) test result was also positive. B and C, The NYC infection rates are based on the percentage of people who tested positive through molecular (RT-qPCR) testing in NYC. The antigen-positive rates represent the percentage of people who tested positive by antigen testing among the total number of people tested*.* The true-positive rates represent the percentage of people who had positive RT-qPCR results among the total number of people tested*.* The false-positive rates represent the percentage of people who had positive antigen test results and negative RT-qPCR test results among the total number of people tested. Additional NYC data were obtained from NYC.gov. The company mandated vaccination for workers onsite in November 2021.

## Discussion

The results of this study demonstrated that when a repeated rapid antigen test was offered to participants of an employee screening program, the estimated accuracy increased from 38% to 92% for true-positive results as determined by RT-qPCR for SARS-CoV-2. These findings may have important implications for how rapid antigen tests can be deployed for more accurate results,^5,6^ especially in a setting where the time to results is important and where widespread PCR testing may be cost prohibitive.

Limitations of the study were that all employees were asymptomatic and were screened as part of a workplace testing program. As expected, test results appeared to be more accurate when community infection rates were higher and, therefore, the pretest probability was higher. The diagnostic value of a second antigen test remained high regardless of pretest probability. As employers consider the best use of onsite or at-home rapid antigen testing, a second antigen test may be useful for more accurate diagnosis of COVID-19 infection and for guiding intervention.
